# Repeated Witnessing of Conspecifics in Pain: Effects on Emotional Contagion

**DOI:** 10.1371/journal.pone.0136979

**Published:** 2015-09-10

**Authors:** Maria Carrillo, Filippo Migliorati, Rune Bruls, Yingying Han, Mirjam Heinemans, Ilanah Pruis, Valeria Gazzola, Christian Keysers

**Affiliations:** 1 Netherlands Institute for Neuroscience, an institute of the KNAW, Amsterdam, The Netherlands; 2 Faculty of Social and Behavioural Sciences, University of Amsterdam (UvA), Amsterdam, The Netherlands; 3 Earth and Life Sciences, VU university, Amsterdam, The Netherlands; State University of New York at Oneonta, UNITED STATES

## Abstract

Witnessing of conspecifics in pain has been shown to elicit socially triggered freezing in rodents. It is unknown how robust this response is to repeated exposure to a cage-mate experiencing painful stimulation. To address this question, shock-experienced *Observer* rats repeatedly witnessed familiar *Demonstrators* receive painful footshocks (six sessions). Results confirm that *Observers* freeze during the first testing session. The occurrence of this behaviour however gradually diminished as the experimental sessions progressed, reaching minimal freezing levels by the end of the experiments. In contrast, the appearance and continuous increase in the frequency of yawning, a behavior that was inhibited by metyrapone (i.e,. a glucocorticoid synthesis blocker), might represent an alternative coping strategy, suggesting that the observer’s reduced freezing does not necessarily indicate a disappearance in the affective response to the *Demonstrator*’s distress.

## Introduction

Empathy, the ability to understand and share the feelings of others, can be conceptualized as a hierarchically organized multi-level capacity such that higher-level processes are built on top of more primal ones, like emotional contagion [1;2]. Emotional contagion is an automatic tendency to converge with another individual’s emotional state as a direct consequence of perception and without distinguishing the origin of the emotion (i.e., self vs other) [3;4]. An increasing amount of evidence suggests that at least the basic components of empathy, namely emotional contagion, are shared with non-human mammals.

Evidence of emotional state sharing in rodents originates from studies showing socially-induced hyperalgesia [5], social priming [6] and social buffering [7,8]. For example, mice and rats learn to fear a conditioned stimulus by simply observing or interacting with a conspecific in distress [6]. Sharing the distress of others has also been evidenced by the socially triggered freezing response exhibited by shock-experienced observers when witnessing demonstrators endure painful foot electroshocks [9–13]. This phenomenon is modulated by the genetic characteristics of the rodent strain [14], context [12] the degree of familiarity between observer and demonstrator and by previous experience of the observers [11]. In addition, the expression of emotional contagion in both mice and humans is dependent on stress levels of the observer animal [15], as reduced affective responses can be restored by administration of a glucocorticoid synthesis inhibitor.

So far, all the studies investigating emotional contagion in rodents have examined the behavior of animals after a single exposure or interaction with another in distress. Despite the increasing characterization of emotional contagion in rodents, it is still unknown how animals would respond following repeated witnessing of a conspecific in pain. How would animals respond following repeated testing with a conspecific in pain? This question is of methodological interest, as many experiments would require repeated testing of an individual, be it to investigate, using repeated measures the effect of drugs or to record neural activity and behavior over multiple exposures. Here, leveraging our rodent model of empathy [9], we exposed shock experienced rats on multiple days to familiar *Demonstrators* undergoing footshocks to investigate whether and how behavior changed from day to day.

## Results and Discussion

**Fig 1:**
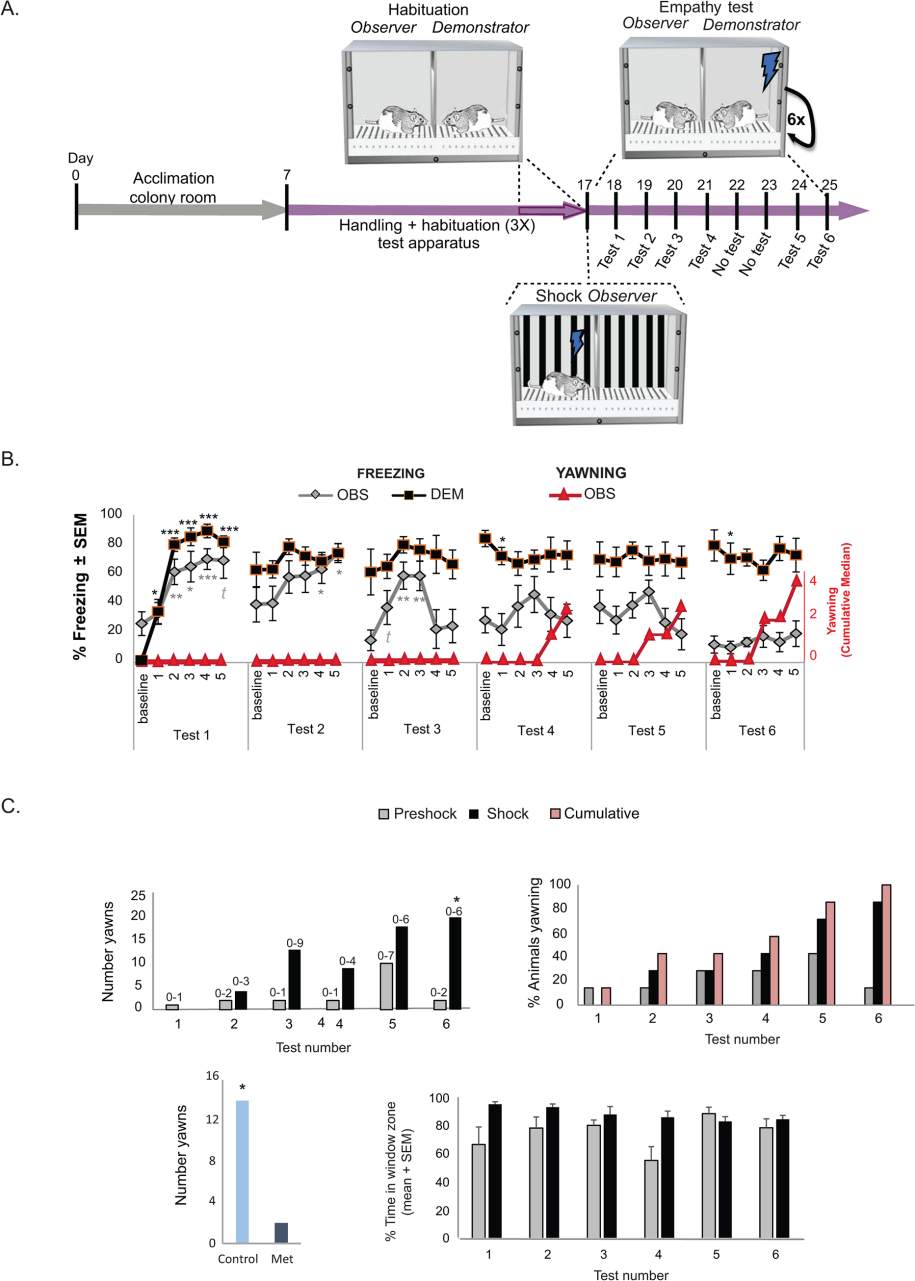
Procedures and results of Experiment 1. (A) Schematic showing the timeline and illustration of the procedures conducted in *Experiment 1*. Following acclimation to the colony room and 7 days of handling, *Observer-Demonstrator* pairs were habituated to the test box (20 minutes/day for 3 days). One day prior to the first empathy test, *Observers* experienced mild shock exposure. Then, *Observer-Demonstrator* pairs experienced 6 semi-consecutive footshock sessions with only 2 days of no test between empathy test 4 and 5. (B) Graph of the freezing responses of *Observers* (gray line) and *Demonstrators* (black line) and yawning of *Observers* (red line). The figure depicts average freezing percent ± standard error of the mean (SEM) of 6 test days (1 to 6) each consisting of a baseline (2 minutes prior to 1^st^ shock) and 5 shock periods: 1^st^ to 2^nd^ shock, 2^nd^ to 3^rd^ shock, 3^rd^ to 4^th^ shock, 4^th^ to 5^th^ shock and 5^th^ plus 2 additional minutes, corresponding to numbers 1 to 5 in the graph. For each test day, freezing percent was compared between time periods where shocks were delivered (i.e., time periods 2, 3, 4, 5 and 6) and baseline period. Black significance symbols are for *Demonstrators* while grey-colored symbols are for *Observers*. In addition, graph shows the cumulative (i.e., daily) median yawning of *Observers* for test days 1 to 6 (red colored y axis on the right side). (C) Top left graph shows the total number of yawns displayed by *Observers*. Comparisons were made between the numbers of normalized yawns during shock period of test days 2 to 6 (normalized to preshock) and normalized yawns during test day 1. Top right graph shows the percent of *Observers* that yawned at least once and the cumulative percent across test days of *Observers* that yawn (pink bars). Bottom left graph shows the total number of yawns over all *Observers* during the shock period (minus the yawns during the preshock period) in the control condition (i.e., no pretreatment with metyrapone) and following a subcutaneous injection of metyrapone (25mg/kg) prior to test. Bottom right graph shows the mean percent of time *Observers* spent in the window zone (i.e., area closest to the separation from the *Demonstrators-*bottom panel). All graphs show results during the preshock (gray) and shock (black) periods of test days 1 to 6. Numbers on top of the bars indicate the range of yawns displayed by the animals. ***p<0.005, **p<0.01, *p<0.05, *t*:*p* = 0.072.

Shock-experienced *Observer* rats were exposed to familiar *Demonstrators* undergoing footshocks during six identical, semi-consecutive testing days. Each testing day contained a preshock baseline period and 5 shocks, during which the freezing of *Demonstrators* and *Observers* was scored (Fig 1A).

### Freezing of Demonstrators

A 6 Days x 6 Epochs (1 baseline + 5 shock periods) ANOVA revealed a significant Day x Epoch interaction (*F*_*25*,*100*_ = 3.96, *p*<0.0001). This interaction was driven by Day 1, as removing Day 1 rendered the interaction non-significant (*p*>0.05). A 6 Day repeated measures ANOVA on baselines showed differences between freezing levels of *Demonstrators* during the baseline period of Days 1 to 6 (*F*_*5*,*30*_ = 10.751, *p*<0.001; Fig 1B). Specifically, relative to the preshock-baseline of Day 1, *Demonstrators* displayed high freezing levels throughout (paired *t*-tests of baseline Day 1 compared to Days 2 to 6, all *p*<0.05). Further, planned comparisons using paired sample *t*-tests of each shock period to baseline, revealed that on Day 1, *Demonstrators* froze more following each one of the five shocks than baseline (all *p*<0.01; Fig 1B). In contrast, the same analysis showed that except for two data points, on Days 2 to 6, the *Demonstrators’* freezing levels during the shock periods was not higher than on baseline. This indicated that after the first test Day *Demonstrators* developed contextual freezing.

### Freezing of Observers

A 6 Days x 6 Epochs ANOVA on the freezing of *Observers* revealed main effects of Days (*F*_5,20_ = 9.45, *p*<0.0001) and Epoch (*F*_5,20_ = 4.1, *p*<0.01; Fig 1B) and a significant Day x Epoch interaction (*F*_5,20_ = 1.68, *p*<0.05). In contrast to *Demonstrators*, a 6 Day repeated measures ANOVA on baselines showed no significant differences between freezing levels during the baseline period of Days 1 to 6 (*p*>0.1). Planned paired sample *t*-tests comparing shock with baseline periods for each Day indicates diminished freezing after Day 4 (Fig 1B). Specifically, *post-hoc t*-tests revealed *Observers* froze significantly more during various shock- periods of Days 1, 2 and 3 (compared to those day’s baseline, all *p*<0.05) while this was not the case on Days 4 to 6 (all *p*>0.4). This reduction in freezing of *Observers*, could suggest that their sensitivity to the distress of the *Demonstrator* diminishes following repeated exposure.

### Emergence of Yawning in Observers

**Fig 2:**
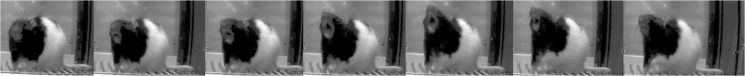
Consecutive frames (< 500 msecs) of an empathy test clip showing yawning of an *Observer* animal during the shock period.

Throughout the experiment, we however noticed the appearance of yawning, an unusual behaviour displayed only by the *Observers* (Fig 2, S1 Video). Yawning included extensive opening of the mouth and most of the times a full-body extension and upward pointing of the snout. A 6 day repeated measures Friedman test on normalized yawns (i.e., number of yawns during the first 10mins of shock period minus number of yawns during preshock period [first 10 minutes at start of test prior to 1^st^ shock]) detected differences in yawning frequency between Days (χ^2^ (5) = 12.12, *p* = 0.033). Planned Wilcoxon *posthoc* tests comparing yawning frequency between Day 1 and Days 2 to 6 revealed that *Observers* yawned more during Day 6 compared to Day 1 (*p* = 0.017). The yawning response of *Observers* appeared to be specific to the distress of the *Demonstrators*, as a Wilcoxon signed rank test comparing the total number of yawns during the combined preshock periods of Days 1 to 6 to the total number of yawns during the shock periods of Days 1 to 6, showed that *Observers* yawned more during the shock periods (*Z*(6) = -2.2, *p*<0.05) (Fig 1B and 1C). Further, the percent of *Observers* yawning increased throughout the experiment, with more than 70% of animals yawning in the last two Days compared with 0% on Day 1 (Fig 1C). Moreover, by Day 6, all *Observers* had yawned at some point indicating a generalized behavioral response. The number of yawns depended on the time of day at which rats were tested. Three rats were tested in the morning (beginning of their dark phase), and showed on average a total of 9 yawns over the 6 days. Four rats were testing in the afternoon (second half of their dark phase) and showed on average a total of 19 yawns. To ensure that the increase of yawns across Days was not due to an inadvertent shift in testing time, the total number of yawns on each Day was compared with the average testing time on that day, but the relationship was clearly non-significant (r^2^<0.0067, p = 0.87). Importantly, the results of a separate experiment showing that yawning was significantly inhibited by pre-treatment with metyrapone (*t*_(18)_ = 2.191, *p* = 0.042), suggests that yawning reflects heightened stress levels in the *Observers* (Fig 1C).

### Attention in *Observers*

To investigate whether the attention of *Observers* towards the Demonstrators changed throughout the experiment, the percent of time *Observers* spent in the window zone (i.e., 12cm x 25cm area closest to the divider from the *Demonstrator’* chamber) was quantified (Fig 1C). A repeated measures ANOVA with 6 Days x 2 Epochs (comparing the 10 minute preshock period prior to shock start and the 10 to 15 minute after the 1^st^ shock) revealed a main effect of Epoch (F_1,4_ = 13.3, *p*<0.05). This indicated that *Observers* were drawn to spend a higher percent of time in the window zone following shock delivery. The absence of an interaction of Epoch and Day (*p*>0.05) however shows this effect to be constant across days, suggesting that the attention of *Observers* was captured by the *Demonstrators* receiving shocks in a way that was sustained throughout the experiment.

### Discussion

*Demonstrators* submitted to repeated and unavoidable foot shocks exhibit elevated freezing levels. Confirming previous findings [9;11;12;16] in the first testing session (i.e., Day 1), shock-experienced *Observers* display freezing in response to familiar *Demonstrators* enduring painful footshocks. As to our core question of whether these effects can be measured over repeated days, as necessary for designs that require repeated testing, our results show that freezing gradually diminishes as a consequence of repeatedly witnessing the *Demonstrator* receive painful stimulations. Results of a separate pilot experiment revealed that this reduction of freezing occurs even if a number of experimental factors are manipulated to reduce such habituation (S1 Fig). Alternating between different testing contexts, increasing the length of time between testing sessions, pairing two *Demonstrators* to each *Observer* and adding a reminder shock session for the *Observers*, failed to avoid a progressive reduction of the *Observer’s* freezing. From an experimental design point of view, our findings thus indicate that socially triggered freezing, as an assay of social sensitivity in rodents may be more suited for between-subject designs (that do not require multiple testing of a given rodent) than for within-subject designs. In addition, at first glance, our findings could suggest that the affective response of *Observers* to the distress of *Demonstrators* is progressively reduced throughout the testing sessions. The appearance of yawning however complicates this interpretation.

Yawning is a phylogenetically old behavior, ubiquitously present across vertebrates [17–21]. It is characterized by an extensive and involuntary opening of the mouth with deep, prolonged inspirations and short expirations lasting approximately 10 seconds and commonly accompanied by stretching. Yawning has been observed in stressful situations in different species like monkeys [22–25], rats [26] and birds [27]. Thus, the emergence of yawning as freezing becomes infrequent could mean that animals change the way they manifest their affective response to the distress of others, but that animals are still responsive to the distress of the other. Yawning as a possible indicator of elevated stress levels in the *Observers* was confirmed by the reduction of this behavior following administration of an anti-stress drug (i.e., metyrapone) on test days 5 and 6. This is in agreement, with results showing that administration of anxiogenic drugs to monkeys induces both anxiety-like behavior and yawning [25], suggesting that indeed yawning is indicative of elevated stress levels. It has been hypothesized that in addition to being a marker of arousal, yawning marks the transition between different types of arousal or levels of arousal [28]. Perhaps then, yawning in *Observers* indicates a change in the type of arousal the *Observers* experience during the first testing days compared to the arousal in the last testing days. In contrast, the repeated daily shock exposure the Demonstrators undergo maintains a highly elevated but similar arousal type throughout the experiment, thus preventing yawning form emerging. However, this is highly speculative and further testing such as corticosterone measurements throughout the experiment would be necessary to confirm this hypothesis. In addition to the postulated role in stress, yawning has been linked to a variety of other functions, such as a thermoregulation [20,29]. Here though all sessions were conducted in conditions of constant room temperature. That yawning appeared following repeated testing, was specific to the shock periods and only present in *Observers* suggests that the most parsimonious explanation for its emergence is that it is indicative of changes in autonomic regulation and reflects an affective response in the *Observer* to the distress of the *Demonstrator*. Together, these findings and the results showing that the *Observers* attention towards the *Demonstrators* is unaltered indicate that although the freezing response to witnessed distress changes over time, the affective response of the *Observers* may outlast socially triggered freezing and invite yawning as a coping mechanism. However, more data will be needed to fully understand the relationship between freezing, yawning and other stress related manifestations.

## Materials and Methods

### Subjects

Male Long-Evans rats (6–8 weeks old/ 250-350g) were obtained from Harlan Laboratories (Germany). Upon arrival animals were socially housed in couples and maintained at ambient room temperature (22–24°C, 55% relative humidity, SPF, type III cages with sawdust), on a reversed 12:12 light-dark cycle (lights off at 07:00). Food and water were provided *ad libitum*. All experimental procedures were preapproved by The Institutional Animal Care and Use Committee of the Netherlands Institute for Neuroscience (IACUC-NIN-1211). Studies were conducted in strict accordance with the European Community’s Council Directive (86/609/EEC).

### Setup

Two adjacent compartments of equal dimensions (each L: 24cm x W:25cm x H:34cm; Med Associates, Inc.) separated by stainless steel bars (6 mm in between bars) which allowed animals to smell, see, hear and touch each other. The compartment walls were made of transparent Plexiglas and the floor of stainless steel grid rods. One of the chambers was connected to a stimulus scrambler (ENV-414S, Med Associates Inc.) for shock exposure of *Observers* and empathy testing. Behavior was video recorded throughout testing using a camera (Model DCRDVD300, Sony Corporation of America, USA) placed ca. 1m in front of the setup onto miniDVDs for behavior scoring.

### Experimental procedures

#### Acclimation, handling and habituation

On arrival day, animals were randomly paired (N = 16, 8 pairs), assigned the role of *Observer* or *Demonstrator* and allowed to acclimate to the colony room for 7 days. Rats were handled during the dark phase of the circadian cycle every other day for 3 minutes for a total of 10 days preceding empathy testing. Three days prior to experiment start, *Observer-Demonstrator* pairs were transported to the experimental room and placed in one of the compartments of the empathy testing apparatus for 20 minutes/day for 3 days. Between each pair, the testing apparatus was cleaned using dishwashing soap and 70% alcohol solution. Throughout the experiment (i.e., habituation and testing, but not pre-exposure) all *Observers* and *Demonstrator*s were always placed in the same compartment of the testing apparatus.

#### Shock pre-exposure

Prior foot-shock experience enhances freezing of *Observers* when witnessing *Demonstrators* experiencing shock [9]. The day preceding empathy testing one of the compartments of the testing box was used to give footshock exposure to *Observers*. To maximize discriminability from the empathy-testing context, the walls were coated with black and white striped paper, the compartment was illuminated with bright white light and cleaned using 30% ethanol solution, followed by dishwashing soap with a neutral smell and 5% vinegar solution. During shock exposure, *Observers were* allowed to acclimate for 10 minutes and then delivered four mild footshocks (0.8mA, 1 sec long, 240–360 sec random inter-shock interval). Compared to the *Demonstrators*, the *Observers* experienced a smaller number and intensity of shocks and a longer intershock interval to minimize the risk of fear conditioning and generalization during empathy testing. The pre-exposure test was conducted during the dark phase of the circadian cycle. *Observer*s were then individually placed in a clean cage for 1 hour prior to return to their home cage.

#### Empathy testing

Thereafter, six daily empathy tests were conducted (Fig 1). For each test, the setup was cleaned using dishwashing soap with neutral smell followed by 70% alcohol solution and illuminated with red dim light. *Observer-Demonstrator* pairs were placed in their respective compartments for a maximum of 30 minutes. After 10 minutes of acclimation, *Demonstrators* received five footshocks (1.5mA, 1 sec each, with random 120–180 seconds inter-shock interval). Following the last shock, animals were left in the testing apparatus for 2 minutes and then returned to their home cage. This procedure was repeated for 4 consecutive days, following 2 days of no test, and then 2 more consecutive test days. The original experimental design included a total of 8 testing days, with 2 resting days in the middle (4 test days, followed by 2 rest days, and lastly 4 test days). However, in this experiment to prevent unnecessary discomfort, the experiment was stopped by test day 6, since the behavior of the animals reached a limit by this day, with *Demonstrators* exhibiting maximal freezing all the time and *Observers* displaying minimal freezing on test days 5 and 6. Testing schedule appears to play a minimal role in the behavior of *Observers*, as different schedules (e.g., 1 week rest in between tests) have no effect on their behavioral response (S1 Fig). All tests were conducted in the dark phase of the circadian cycle, between 08:30 and 16:30. Testing order was constant throughout days, i.e. the rats that were tested first on day 1 were tested first on all other days. Because testing time is known to influence the number of yawns shown by animals [30] it is important to note, that we used within subject statistics to look at differences across testing days, and that the systematic difference in testing time across animals are partitioned out in these statistics as between subject variance, and do not flow into the effect of test day.

### Behavior scoring

Three experienced researchers manually scored the behavior exhibited by both *Observers* and *Demonstrators* during all empathy tests (inter-rater reliability assessed with Pearson’s r correlation coefficient was > 90%). Freezing behavior was defined as lack of movement (except movement due to breathing) for a period longer than 3 seconds. Every empathy test session was subdivided in two different periods: pre-shock and shock. The first period (pre-shock), corresponded to first 10 minutes immediately after the animals were placed in the test apparatus. The second period (shock) was further subdivided into five time bins: (1) from minute 10 to the first shock (baseline period), (2) from the 1^st^ to the 2^nd^ shock, (3) from the 3^rd^ to the 4^th^ shock, (4) from the 4^th^ to the 5^th^ shock and (5) after the 5^th^ shock 2 minutes were added. For each period and time bin, freezing was scored as the percentage of time the animal spent freezing.

Yawning was defined as a wide opening of the mouth sometimes accompanied by stretching and elongation of the body (Fig 2, S1 Video). The yawning frequency of *Observers* was scored throughout all tests. Since the occurrence of yawning was low, the frequency of this behavior was scored for 10 minutes following the 1^st^ shock rather than individually for each intershock interval. To have a baseline period of a comparable length, yawning was scored during the first ten minutes prior to shock delivery. Lastly, the percent of time from the total test time that *Observer* animals spent in the area closest to the *Demonstrator’s* compartment (window zone- 12cm x 25cm area) was scored during the pre-shock and shock periods.

### Statistics

The freezing responses of *Observers* and *Demonstrators* in *Experiment* 1 were analyzed separately using a 2-way repeated measures analysis of variance (ANOVA). The factors examined included test day (1 to 6) and Epoch (i.e., 2 minutes baseline, 1^st^ to 2^nd^ shock, 2^nd^ to 3^rd^ shock, 3^rd^ to 4^th^ shock, 4^th^ to 5^th^ shock and 5^th^ shock plus 2 minutes) as the within factors. All *posthoc* analysis on freezing responses were conducted by using planned contrasts: (1) baseline of Day 1 was compared to baseline of Days 2 to 6 and (2) for each test day, freezing during each shock epoch (1 to 5) was compared to the baseline epoch. One pair was excluded of the analysis because the *Demonstrator* did not show any reaction to any of the shocks in two consecutive sessions.

For the analysis of yawning by *Observers* the test was divided into two Epochs: (1) the preshock/baseline (i.e., first 10 minutes prior to shock onset) and (2) shock period (i.e., 10 minutes following delivery of 1^st^ shock). To investigate general differences between preshock and shock periods, the median yawning frequency was compared using Wilcoxon rank signed test across all test days. The frequency of yawning was normalized to the preshock period, which was achieved by subtracting the preshock frequency from the value observed during the entire shock period. Friedman rank sum test was used to examine differences between test days (repeated measures) in the yawning frequency. Post-hoc analyses were conducted by using planned comparisons between the first test day and all other test days. For the analysis of time spent in the window zone by *Observers* the test was divided into two Epochs: (1) the preshock (i.e., 10 minutes prior to shock start) and (2) shock period (i.e., from delivery of 1^st^ shock until 2 minutes past the 5^th^ shock, for a total time of 10–15 minutes). The percent of time *Observer* animals spent in the window zone was analyzed using a two way repeated measures ANOVA, with Day and Epoch (preshock vs shock) as the within factors.

### Control experiment with Metyrapone

The glucocorticoid synthesis inhibitor, metyrapone (2-metyl-1,2-di-3-pyridyl-1-propanone; Sigma Aldrich) was used to investigate the effects of stress on yawning. For this experiment, a separate set of male Long-Evans rats (n = 19, 6–8 weeks upon arrival) obtained from Janvier (France) was used. The overall experimental procedures including housing, acclimation, handling, habituation, pre-exposure and test were similar to those described above with some modifications. First, test time was between 08.30am to 12.30pm and testing order was randomized daily, with each animal tested at a different time each test day. Second, since these animals were part of an ongoing study that tested effects of familiarity on affective responses of *Observers*, their familiarity with their *Demonstrator* varied based with 1,3 or 5 weeks of paired caging. Importantly however, no significant differences were observed in either the freezing levels or yawning depending on familiarity, allowing us to group the results. Following test days 1 to 4, animals were randomly split in two groups (each familiarity level was split in two halves). The first group received a pretreatment with metyrapone on test day 5 and then underwent a normal test on day 6, while the second group experienced normal testing conditions on test day 5 and received the pretreatment with metyrapone on test day 6. Metyrapone was delivered to all animals 30 minutes prior to start of test. All animals received a 25mg/kg subcutaneous injection of Metyrapone (half-life of 1.9 ± 0.7 hours), which was prepared by dilution in 0.9% saline solution. The number of yawns during the preshock and shock period of test days 5 and 6 were quantified. Then, for statistical analysis, a paired two-tailed *t-test* on the normalized number of yawns (i.e., yawns during shock minus yawns during preshock) following metyrapone injection was compared to the number of yawns following no injection.

## Supporting Information

S1 ARRIVE Checklist Arrive guidelines checklist for animal research showing the section of the manuscript where each recommended item in the checklist is located. (PDF)

S1 Fig Procedures and results of pilot study. This pilot was conducted to examine if manipulation of various experimental factors (e.g., context) would extend the freezing response of *Observers*. For this purpose various changes were implemented. First, an additional *Demonstrator* was paired to each *Observer*. Second, *Observers* experienced an extra shock-exposure session halfway through the experiment. Third, the number of days in-between each empathy test was increased. Fourth, for extra habituation, *Observer-Demonstrator* pairs were placed on a second testing setup (identical to the first one) 30 minutes prior to each test and lastly; three different contexts (represented by green, blue and red color) were used in addition to the *Observers* shock exposure context. (A) Schematic showing the timeline and illustration of the procedures conducted. On arrival day, animals (N = 24) were randomly distributed to one of eight groups, each group consisting of three animals (one *Observer* and two *Demonstrators- DEM1;* black color-coated rat and *DEM2;* brown color-coated rat). Acclimation and handling procedures were identical to those detailed for *Experiment 1*. In contrast to *Experiment* 1, three habituation sessions were conducted prior to each pair of tests. For each habituation session (triangles), *Observers* were first habituated with *DEM1* and then with *DEM2* (20 minutes/each). *Observer* animals experienced two shock exposure procedures (same as in *Experiment 1*), one the day before the first empathy test and the second one after the fourth empathy test. Each *Observer* experienced four alternating empathy tests (Test 1–8) with each one of the *Demonstrators* of their group for a total of eight empathy tests per *Observer*. Three different contexts were used throughout the tests. For Context A the test box was cleaned with dishwashing soap with neutral smell and then 70% ethanol, the setup was illuminated with red dim light and *Observers* were placed in the right compartment (tests 1, 2, 7 and 8). For Context B, the test box was cleaned with hand washing soap with lemon smell and 30% vinegar, the walls were coated with black and white stripes printed paper, the setup was illuminated with white bright light and *Observers* were placed in the left compartment (tests 3 and 4). Lastly for Context C, the test box was cleaned with dishwashing soap with neutral smell, followed by application of rose oil, the walls were coated with white paper, the setup was illuminated with red light and *Observers* were placed in the right compartment (tests 5 and 6). Each empathy testing was conducted as described for *Experiment 1* and pairs were left in a clean cage for 1 hour prior to returning to their home cage. (B) Graph of the freezing responses of *Observers* (gray line) and *Demonstrators* (black line) and yawning of *Observers* (red line). Graph depicts average freezing percent ± standard error of the mean (SEM) of 8 test days (1 to 8) each consisting of a baseline (2 minutes prior to 1^st^ shock) and 5 shock periods: 1^st^ to 2^nd^ shock, 2^nd^ to 3^rd^ shock, 3^rd^ to 4^th^ shock, 4^th^ to 5^th^ shock and 5^th^ plus 2 additional minutes, corresponding to numbers 1 to 5 in the graph. Black significance symbols are for *Demonstrators* while grey-colored symbols are for *Observers*. The background color in the rats test box represents the context in which the test was conducted. In addition, graph shows the cumulative (i.e., daily) median yawning of *Observers* for test days 1 to 8 (red colored y axis on the right side). Two-way ANOVA results of the *Demonstrators* freezing revealed significant main effects in test day (*DEM1*: *F*_*3*,*18*_ = 13.889, *p*<0.001; *DEM2*: *p*>0.05) and time period (*DEM1*: *F*_*5*,*30*_ = 23.37, *p*<0.001; *DEM2*: *F*_*5*,*30*_ = 21.79, *p*<0.001) as well as significant interaction between test day and time period (*DEM1*: *F*_*15*,*90*_ = 2.67, *p*<0.005 and *DEM2*: *F*_*15*,*90*_ = 2.976, *p*<0.001). With one exception (baseline of test 1 compared to baseline of test 7, *p*<0.05), baseline levels of all testing days were comparable to test 1 (*p*>0.05). Also, *Demonstrators* froze significantly less (tests 1, 2, 3 and 4) or approximating significance (tests 5, 6 and 7) during the baseline period compared to shock periods of all tests except test 8. Significant main effects in the *Observers* freezing were detected between test days (*F*_7,35_ = 11.25, *p*<0.001) and time periods (*F*_5,25_ = 6.5, *p*<0.001), but no effect in their interaction (*p*>0.05). *Observers* displayed lower freezing responses during the baseline of tests 3, 6, 7 and 8 and during the shock periods of tests 5, 6, 7 and 8 compared to the first and second testing day (*p*<0.05 for all comparisons). (C) Top left graph shows the total number of yawns displayed by *Observers*. A Friedman test revealed significant differences in normalized yawning between test days (χ^2^ (7) = 15.2, *p*<0.05). Posthoc comparisons (Wilcoxon) were made between the numbers of normalized yawns during shock period of test days 2 to 6 (normalized to preshock) and normalized yawns during test day 1.These analysis showed higher number of yawns on test days 4 (*p*<0.05) and 8 (*p* = 0.066) compared to test day 1. Top right graph shows the percent of *Observers* that yawned at least once on each test day and the cumulative percent across test days of Observers that yawn (pink bars). All graphs show results during the preshock (gray) and shock (black) periods of test days 1 to 8. Numbers on top of the bars indicate the range of yawns displayed by the animals. *p<0.05, **p<0.01, ***p<0.005, *t*: *p*<0.07.
(EPS)

S1 Video Short video clip with an *Observer* animal (right side of the cage) displaying a yawn. (MP4)
